# Epigenetic Analysis of Circulating Tumor DNA in Localized and Metastatic Prostate Cancer: Evaluation of Clinical Biomarker Potential

**DOI:** 10.3390/cells9061362

**Published:** 2020-05-31

**Authors:** Marianne Trier Bjerre, Maibritt Nørgaard, Ole Halfdan Larsen, Sarah Østrup Jensen, Siri H. Strand, Peter Østergren, Mikkel Fode, Jacob Fredsøe, Benedicte Parm Ulhøi, Martin Mørck Mortensen, Jørgen Bjerggaard Jensen, Michael Borre, Karina D. Sørensen

**Affiliations:** 1 Department of Molecular Medicine, Aarhus University Hospital, 8200 Aarhus N, Denmark; mtbjerre@clin.au.dk (M.T.B.); mno@clin.au.dk (M.N.); ohl@clin.au.dk (O.H.L.); soj@clin.au.dk (S.Ø.J.); siri.strand@clin.au.dk (S.H.S.); jcf@clin.au.dk (J.F.); 2 Department of Clinical Medicine, Aarhus University, 8200 Aarhus N, Denmark; bjerggaard@skejby.rm.dk (J.B.J.); borre@clin.au.dk (M.B.); 3 Department of Urology, Aarhus University Hospital, 8200 Aarhus N, Denmark; Martin.Moerck.Mortensen@vest.rm.dk; 4 Department of Urology, Regional Hospital West Jutland, 7500 Holstebro, Denmark; 5 Department of Urology, Herlev andGentofte Hospital, 2730 Herlev, Denmark; peter.busch.oestergren@regionh.dk (P.Ø.); mikkelfode@gmail.com (M.F.); 6 Department of Pathology, Aarhus University Hospital, 8200 Aarhus N, Denmark; beneulho@rm.dk

**Keywords:** prostatic neoplasms, DNA methylation, liquid biopsy, circulating tumor DNA, biomarkers

## Abstract

Novel and minimally-invasive prostate cancer (PCa)-specific biomarkers are needed to improve diagnosis and risk stratification. Here, we investigated the biomarker potential in localized and de novo metastatic PCa (mPCa) of methylated circulating tumor DNA (ctDNA) in plasma. Using the Marmal-aid database and in-house datasets, we identified three top candidates specifically hypermethylated in PCa tissue: *DOCK2,* *HAPLN3,* and *FBXO30* (specificity/sensitivity: 80%–100%/75–94%). These candidates were further analyzed in plasma samples from 36 healthy controls, 61 benign prostatic hyperplasia (BPH), 102 localized PCa, and 65 de novo mPCa patients using methylation-specific droplet digital PCR. Methylated ctDNA for *DOCK2/HAPLN3/FBXO30* was generally not detected in healthy controls, BPH patients, nor in patients with localized PCa despite a positive signal in 98%–100% of matched radical prostatectomy tissue samples. However, ctDNA methylation of *DOCK2,* *HAPLN3,* and/or *FBXO30* was detected in 61.5% (40/65) of de novo mPCa patients and markedly increased in high- compared to low-volume mPCa (89.3% (25/28) vs. 32.1% (10/31), *p* < 0.001). Moreover, detection of methylated ctDNA was associated with significantly shorter time to progression to metastatic castration resistant PCa, independent of tumor-volume. These results indicate that methylated ctDNA (*DOCK2/HAPLN3/FBXO30*) may be potentially useful for identification of hormone-naïve mPCa patients who could benefit from intensified treatment.

## 1. Introduction

Prostate cancer (PCa) is the most common non-cutaneous male cancer in western countries and constitutes a major health challenge. Staging of PCa at diagnosis is crucial for risk stratification and treatment selection as it reflects the size and location of the cancer and presence of possible metastases. Localized PCa is often treated with curative intent using radical prostatectomy (RP) or radiation therapy (RT). Patients presenting with metastatic disease at diagnosis (de novo metastatic PCa) and patients who experience non-localized recurrence after RP or RT are routinely treated with androgen deprivation therapy (ADT). Recently, docetaxel, abiraterone acetate (Zytiga), and apalutamide (Erleada) have been approved for combination therapy with ADT due to improved progression-free and overall survival [1,2,3,4,5,6]. However, the selection of patients for combination therapy is poorly qualified by the current guidelines, which depend on image-based assessment of the tumor volume [2,3]. Thus, novel biomarkers for prognostic risk stratification are urgently needed to guide treatment decisions in metastatic PCa (mPCa).

Candidate DNA methylation biomarkers for PCa with diagnostic and prognostic potential have previously been identified by analyses of tumor tissue specimens [7,8,9,10,11,12,13,14,15]. Tissue from localized PCa is relatively easy to obtain from e.g., RP specimens, whereas attaining tissue biopsies from mPCa is challenging due to the predominant spread to bones. Furthermore, multifocality and the heterogeneous nature of primary PCa complicate the use of tissue biopsies, as undersampling of the tumor may mislead the evaluation [16,17]. One possible way to overcome these issues is by using liquid biopsies to evaluate circulating tumor DNA (ctDNA) from plasma samples. In patients with de novo metastatic or metastatic castration-resistant PCa (mCRPC), genomic profiles of ctDNA in plasma have been shown to closely mirror the profile of matching tumor/metastasis tissues [18,19]. Accordingly, ctDNA has been studied extensively in the past years for its potential use as a genomic biomarker to guide treatment selection in mCRPC [20,21,22,23]. However, until now, few studies have explored the clinical potential of ctDNA methylation markers in plasma from PCa patients and the focus has primarily been limited to *GSTP1* [24,25,26,27,28,29,30].

In this study, we aimed to identify and validate potential ctDNA methylation biomarkers and evaluate their quantity and biomarker potential for risk stratification in a clinical cohort comprising plasma from healthy controls and patients with benign prostatic hyperplasia (BPH), localized PCa, and de novo mPCa.

## 2. Materials and Methods

### 2.1. External 450K Methylation Data (Marmal-Aid)

Infinium HumanMethylation 450 (450K) data was downloaded from the publicly available Marmal-aid database [31]. The dataset consists of more than 14,000 human tissue/blood samples, of which 4047 are male samples with known disease stage and tissue type. In brief, the male samples consisted of 876 peripheral blood cell (PBC) samples, 81 normal prostate tissue samples, 187 PCa tissue samples, 598 normal tissue samples (other than prostate), and 2042 tissue samples from 14 other cancer types (e.g., colon, bladder, kidney, and lung). DNA methylation was reported as β-values, ranging from 0 (unmethylated) to 1 (fully methylated).

### 2.2. In-House Patient Samples

Patient samples were obtained at Aarhus University Hospital, Regional Hospital West Jutland, and Herlev-Gentofte Hospital between 1999 and 2017.

For qMSP analyses (small-scale experimental evaluation), formalin-fixed paraffin-embedded (FFPE) RP tissue specimens from 20 patients with histologically verified localized PCa were available. Non-PCa controls included adjacent normal (AN) FFPE RP tissue from 13 PCa patients with localized disease, FFPE transurethral resection of the prostate (TUR-P) tissue samples from 7 patients with BPH, 20 whole-blood samples from patients with localized PCa, and 20 buffy-coat samples from healthy male blood donors (i.e., mainly leukocytes, PBC) (Table S1). Four RP tissue samples and one BPH tissue sample were excluded due to insufficient DNA amounts after bisulfite conversion.

The sample set used for technical evaluation of methylation-specific droplet digital PCR (MS-ddPCR) assays (designed for selected candidate genes) included PBC (buffy coat) samples from 52 healthy male blood donors and EDTA-plasma from 70 healthy individuals (7 women/63 men, Table S1). Eight PBC samples and six samples from healthy individuals were excluded due to insufficient DNA amounts before or after bisulfite conversion. Regarding EDTA-plasma samples from healthy individuals, samples from 16 patients were used for each of four assays.

The clinical cohort (analyzed by MS-ddPCR) consisted of EDTA-plasma samples from 63 patients with BPH, 102 patients with histologically verified localized PCa, 66 patients with mPCa, and 36 healthy male blood donors (Table 1). Plasma samples from two mPCa patients were excluded due to bisulfite conversion failure. Additionally, two BPH patients were reclassified as de novo metastatic PCa, however, one of these patients had received ADT prior to sampling and was excluded from the mPCa group in the final analyses. For EDTA-plasma samples from healthy individuals, samples from 12 individuals were used for each of three MS-ddPCR assays.

Finally, MS-ddPCR was used to further analyze a subset of patients from the clinical cohort with matched PCa tissue and plasma samples available. This set consisted of FFPE RP tissue specimens and EDTA-plasma samples from 55 patients (Table S1). Fourteen tissue samples were excluded due to insufficient DNA amounts. The final sample set consisted of 41 matching RP tissue and EDTA-plasma samples.

In all cases, blood samples were collected immediately prior to surgery (RP/TUR-P). For de novo mPCa patients, plasma was drawn at diagnosis prior to treatment.

### 2.3. DNA Isolation from Tissue and Blood

DNA was extracted from FFPE TUR-P specimens (BPH) and FFPE punch biopsies from RP specimens (PCa and AN), according to the standard protocol for the QIAamp DNA FFPE Tissue Kit (Qiagen, Hilden, Germany), as described previously [12]. DNA from PBC samples was extracted from whole blood or buffy coat using the QiaSymphony DSP DNA Midi Kit (Qiagen).

Whole blood was collected in BD Vacutainer K_2_ EDTA tubes (Beckton Dickinson, Franklin Lakes, NJ, USA) and processed within 4 h, as described below. Blood samples from PCa patients and controls were processed in the same way. To separate plasma from cellular components, EDTA-blood samples were centrifuged at 3000*g* for 10 min at 20 °C, and plasma was stored in cryo tubes (TPP) at −80 °C until DNA extraction (<5 years). Plasma samples were thawed at room temperature, centrifuged at 3000× *g* for 10 min at 20 °C, and circulating cell-free DNA (cfDNA) from 4–20 mL of plasma was extracted on a QIAsymphony robot using the QIAamp Circulating Nucleic Acids kit (Qiagen), as specified by the manufacturer. Extracted cfDNA was eluted in LoBind tubes (Eppendorf AG, Hamburg, Germany) and stored at −80 °C until further use (<16 months). Extraction efficiency (Table S2) and potential contamination with genomic DNA from lysed peripheral blood mononuclear cells (PBMCs) were assessed by ddPCR, as previously described [32]. In brief, a fixed amount of soybean CPP1 DNA fragments was added to each plasma sample prior to cfDNA extraction. Extraction efficiency was calculated as the percent recovery of CPP1 fragments spiked in prior to cfDNA extraction (CPP1 ddPCR assay). PBMC DNA contamination was estimated by a ddPCR assay targeting the VDJ rearranged IGH locus, specific for B lymphocytes (PB assay). The median extraction efficiency was 79% (interquartile range 66%–94%). Contamination with PBMC DNA was observed in less than 4% of all plasma samples (12/335) and since cfDNA concentrations did not deviate from the rest of the samples and were not removed. Before bisulfite conversion, each cfDNA sample was dried using vacuum centrifugation (SpeedVac, Concentrator plus 5350, Eppendorf AG) at 30 °C, resuspended in 20 µL AccuGENE Molecular Biology Water (Lonza, Basel, Switzerland), and bisulfite converted as described below.

### 2.4. Bisulfite Conversion

Genomic and cell-free DNA samples were bisulfite converted using the EZ-96 DNA Methylation-Direct™ kit (Zymo Research, Irvine, CA, USA), according to the manufacturer’s instructions. Briefly, methylated and unmethylated DNA standards (Zymo Research) were included in each bisulfite conversion batch as positive and negative controls, and samples were incubated on a S1000 Thermal cycler (Bio-Rad, Hercules, CA, USA). The bisulfite converted DNA extracted from tissue and PBCs was stored for up to 2 weeks prior to quantitative methylation-specific PCR (qMSP) analysis. The bisulfite converted cfDNA was analyzed by MS-ddPCR immediately after completed bisulfite conversion (Table S2).

### 2.5. Quantitative Methylation-Specific PCR (qMSP)

qMSP assays were designed using Beacon Designer (Premier Biosoft, Palo Alto, CA, USA). Primer and probe sequences are provided in Table S3. qMSP reactions were run in triplicates using 5 μL Taqman Universal Mastermix no UNG (Applied Biosystems, Waltham, MA, USA), 5 ng bisulfite-converted DNA, 5–12 pmol of each primer, and 2–4 pmol probe in a total volume of 10 µL. On each plate, seven-point serially diluted methylated DNA (bisulfite converted CpGenome Universal Methylated DNA (Merck Millipore, Burlington, MA, USA)) and two negative controls (H_2_O and whole-genome amplified (WGA) DNA) were included. *ALUC4* and *MYOD1* were used for quality control and normalization (*ALUC4*). Reactions were run on the ViiA7 Real-Time PCR System (Applied Biosystems) in 384-well plates: 2 min at 50 °C, 10 min at 95 °C, and 40 cycles of 15 s at 95 °C and 1 min at 56–60 °C (Table S3). Quantities were estimated from the standard curves using QuantStudio™ Real-Time PCR Software (Applied Biosystems). Outliers (>2 Ct (cycle threshold) values lower or higher than the other replicates) and samples with *ALUC4* Ct > 24.0 in ≥ 2 of 3 replicate reactions and/or without a *MYOD1* positive signal were removed. Samples were considered negative for methylation if ≥2 methylation-specific reactions did not amplify.

### 2.6. Methylation-Specific Droplet Digital PCR (MS-ddPCR)

MS-ddPCR primers and probes were designed to be exclusively specific for methylated bisulfite-converted DNA. Locked nucleic acids (LNA™) were incorporated into primers and probes to increase assay specificity and reduce amplicon lengths (Table S4) [32,33].

Samples were analyzed on the QX200 Droplet Digital PCR System (Bio-Rad) according to the manufacturer’s instructions and performed in accordance with the Minimum Information for Publication of Quantitiative Digital PCR Experiments (dMIQE) guidelines [34] (Table S5).

The ddPCR reaction mastermix was prepared as follows: 2–9 µL template DNA, 18 pmol forward primer, 18 pmol reverse primer, 5 pmol probe, 11μL 2xSupermix for Probes no UTP (Bio-Rad), and 0–7 μL AccuGENE™ Molecular Biology Water (Lonza) to a final volume of 22 µL. One nanoliter droplets were generated on the QX200 Automated Droplet Generator (Bio-Rad). The median number of droplets was 15,973 (interquartile range 14,750–16,981). After droplet generation, samples were amplified by PCR in a S1000 Thermal cycler (Bio-Rad) at (i) 95 °C for 10 min, (ii) 45 cycles of 95 °C for 30 s and 56–60 °C for 1 min, and (iii) 98 °C for 10 min. Amplified samples were stored at 4 °C for up to 12 h before analysis on the QX200 reader (Bio-Rad). Positive and no-template controls were included for each assay on each plate. For methylation-specific assays, an unmethylated negative control was also included. Quantasoft v1.7 software (Bio-Rad) with standard settings was used for analysis of ddPCR data.

### 2.7. cfDNA Quantification before and after Bisulfite Conversion

Extracted cell-free DNA was quantified by ddPCR using assays targeting two reference regions located on chromosome 1 (CF assay) and chromosome 3 (Chr3 assay), respectively, as previously described [35]. Both assays are located in regions that only rarely show copy number alterations in cancer, including PCa. Reported quantities are the average of the two assays. The CF assay was designed to amplify a cytosine-free region of the genome, thereby enabling the use of the same assay for quantification of both native and bisulfite converted DNA. The CF assay was used for DNA quantification and recovery assessments after bisulfite conversion. The recovery was calculated as the CF quantity after bisulfite conversion divided by the CF quantity of native DNA.

### 2.8. Statistical Analysis

All statistical analyses were conducted in R studio version 3.5.0. Mann–Whitney tests were applied to investigate differences between groups. When appropriate, *p*-values were adjusted using the Benjamini–Hochberg method to correct for multiple testing [36]. Spearman’s correlation tests were used to investigate associations between cfDNA or ctDNA and disease stages as well as clinicopathological parameters. Receiver operating characteristic (ROC) curves were used to evaluate sensitivity and specificity. For survival analyses, Kaplan–Meier curves and Cox regression analyses were used with progression to mCRPC, PCa-specific survival, or overall survival (OS) as endpoints. Six mPCa patients were excluded from survival analyses due to missing follow-up or because they had received chemotherapy in combination with ADT at mPCa diagnosis. Thus, a total of 59 and 26 mPCa patients were used in the survival analyses for *DOCK2*/*HAPLN3* and *FBXO30*, respectively.

### 2.9. Ethics Approval and Consent to Participate

The Central Denmark Region Committees on Health Research Ethics and the Danish Data Protection Agency approved the study. All patients provided written informed consent.

## 3. Results

### 3.1. Identification of PCa-Specific DNA Methylation Biomarker Candidates Suitable for Blood-Based Analyses

**Figure 1:**
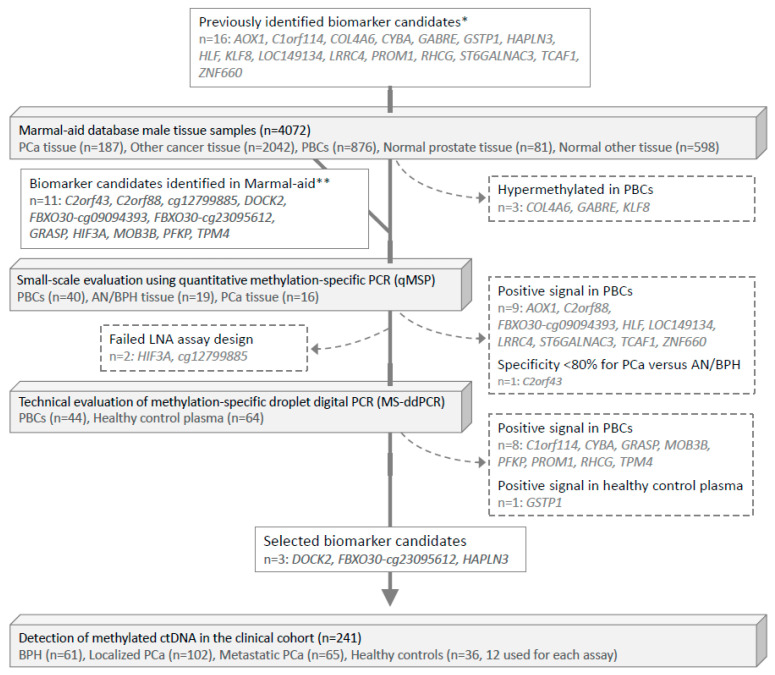
Flowchart of biomarker selection and validation. PCa, prostate cancer. PBCs, peripheral blood cells. AN, adjacent normal. BPH, benign prostatic hyperplasia. * Biomarker candidates were identified in the following previous studies: Strand et al. [12] (*COL4A6, CYBA, HLF, LOC149113, LRRC4, PROM1, RHCG, TCAF1*); Haldrup et al. [10] (*AOX1, C1orf114, HAPLN3, KLF, ST6GALNAC3, ZNF660*); Kristensen et al. [7] (*GABRE*); and Goering et al. [26] (*GSTP1*). ** Bjerre et al. [14].

First, the Marmal-aid database [31] was used for in silico evaluation of the risk of false positive (PBC-derived) methylation signals in blood (plasma) for 16 biomarker candidates previously reported as hypermethylated in PCa vs. normal prostate tissue samples [7,10,12,30] (Figure 1). Here, three of these markers (*COL4A6*, *GABRE*, *KLF8*) were excluded as they showed hypermethylation in PBCs (Figure 1 and Figure S1). Additionally, a set of 11 PCa-specifically hypermethylated biomarker candidates (previously identified from the Marmal-aid database [14]) were also included (Figure 1 and Figure S1). Thus, for the present study, a total of 24 biomarker candidates significantly hypermethylated in PCa tissue (*n* = 187) compared to PBC samples (*n* = 876), normal prostate tissue samples (*n* = 81), tissue samples from 14 other cancer types (*n* = 2042), and other normal tissue samples (*n* = 598) were selected for further evaluation of their PCa-biomarker potential in plasma (*p* < 0.001, Mann–Whitney test, Figure S1).

Next, for small-scale experimental validation by qMSP, we measured the methylation levels of these 24 candidate markers in a distinct set of 40 PBC samples from healthy donors, 19 AN/BPH tissue samples, and 16 PCa tissue samples (small-scale evaluation, Figure 1 and Figure S2). Ten candidates were excluded due to false-positive signals in PBCs or suboptimal specificity for PCa (Figure S1 and Table S3).

**Figure 2:**
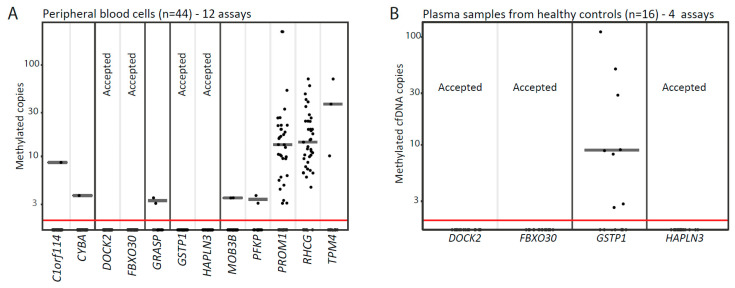
Evaluation of biomarker candidates using methylations-specific droplet digital PCR. (**A**) Twelve methylation-specific droplet digital PCR (MS-ddPCR) assays were tested on DNA from peripheral blood cell samples from healthy donors (*n* = 44). (**B**) Four MS-ddPCR assays were further tested on cfDNA samples from healthy donors (*n* = 16). Each dot represents one analyzed sample. Candidate genes with no signal are marked with Accepted. Median values are indicated with horizontal grey lines. cfDNA, circulating cell-free DNA.

Out of the remaining 14 candidates, we were able to design LNA-based MS-ddPCR assays for 12 candidates (Figure 1). For technical evaluation of the specificity of these assays, we analyzed genomic DNA from a total of 44 PBC and 64 cfDNA (plasma) samples from healthy donors (Figure 2A,B). This led to the exclusion of another 9 candidate genes, due to detection of false-positive signals (Figure 1). Finally, the remaining three top candidate methylation markers (*DOCK2*, *FBXO30-cg23095612*, and *HAPLN3*; Table S6) were selected for large-scale evaluation in the clinical cohort (*n* = 241), as they showed no false-positive signals and also had high sensitivity/specificity for PCa in the technical validation phase (Figure 1).

### 3.2. CfDNA Abundance in the Clinical Cohort

In our clinical cohort (Table 1), the cfDNA concentration (copies/mL plasma) varied widely, but there were no significant differences between healthy blood donors (median 1814 (range: 629–3116)), BPH patients (median 1811 (range: 827–10823), localized PCa (median 1693 (range: 593–14321)), or de novo mPCa patients (median 1966 (range: 599–59500)) (Figure S3A, *p* > 0.05, Mann–Whitney). Moreover, in the localized PCa patient group, cfDNA concentration did not correlate with serum PSA levels, Gleason Grade Group, or N-stage (*p* = 0.218, > 0.05, > 0.05, respectively. Figure S3B–D), but patients with higher clinical stage (cT3–4 vs. cT1–2) had significantly higher cfDNA concentrations (Figure S3E. *p* = 0.018, Mann–Whitney). However, the absolute difference was not large enough to clearly separate these two groups (Figure S3E). Similarly, in patients with de novo mPCa, clinical T-stage was the only clinicopathological parameter significantly associated with cfDNA concentration (*p* = 0.030, Figure S3F). Thus, in the present patient set, higher plasma cfDNA concentration was associated with more advanced clinical T-stage but not with any of the other clinicopathological parameters.

### 3.3. Detection of Methylated ctDNA in Plasma Samples from Patients with Localized PCa

Due to limited amounts of plasma available, only two of the three selected biomarker candidates could be examined in the entire clinical cohort consisting of 228 patients (61 BPH, 102 localized PCa, 65 mPCa) and 12 healthy controls (Figure 1, Table 1). *DOCK2* and *HAPLN3* were selected for full examination owing to the previously demonstrated prognostic value of these DNA methylation biomarkers in PCa tissue samples [10,14]. Additionally, *FBXO30-cg23095612* (from now on, this will be referred to as *FBXO30*) was evaluated in plasma samples from 81 patients (36 BPH, 13 localized PCa, 32 mPCa) and 12 healthy controls.

**Figure 3:**
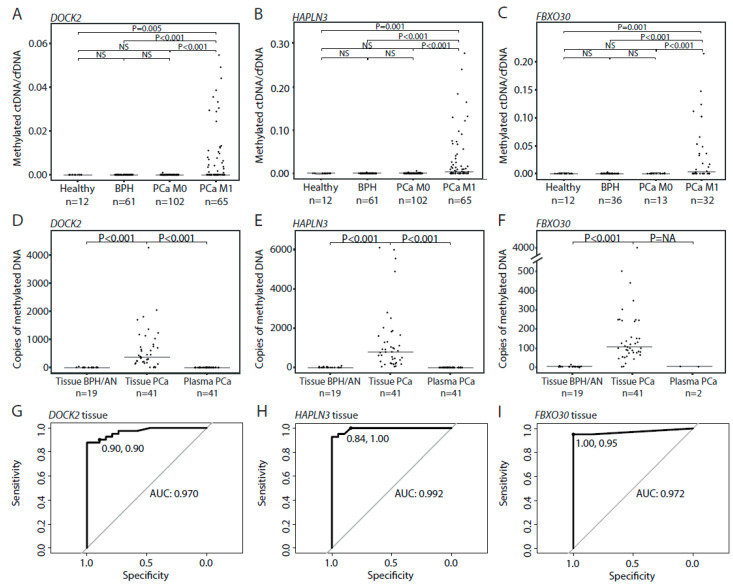
Circulating tumor DNA (ctDNA) detection in plasma and matched PCa tissue samples using MS-ddPCR. (**A**–**C**) Ratio between methylated ctDNA and cfDNA copies for *DOCK2* (**A**), *HALPN3* (**B**), and *FBXO30* (**C**) in plasma samples from healthy controls, BPH, localized PCa (PCa M0), and metastatic PCa patients (PCa M1). Each dot represents one analyzed sample. (**D**–**F**) Total number of copies of *DOCK2* (**D**), *HALPN3* (**E**), and *FBXO30* (**F**) in BPH/AN tissue samples, and in matched PCa tissue samples and plasma samples from patients with localized PCa. Each dot represents one analyzed sample. (**G**–**I**) ROC curves comparing 19 BPH/AN and 41 PCa tissue samples for *DOCK2* (**G**), *HALPN3* (**H**), and *FBXO30* methylation (**I**). *p*-values were calculated using Mann–Whitney tests. Horizontal bars indicate median values in each group. Healthy, healthy controls. BPH, benign prostatic hyperplasia. PCa, prostate cancer. M0, non-metastatic disease. M1, metastatic disease. ROC, receiver operating curve. AUC, area under the curve.

Methylated ctDNA was not detected in plasma from BPH patients with the *DOCK2* assay (0/61) but was detected in one patient with the *FBXO30* (1/36; 2.8%) and the *HAPLN3* assays (1/61; 1.6%) (Figure 3A–C). Likewise, methylated ctDNA was detected in plasma from only one patient with localized PCa with the *DOCK2* (1/102; 1.0%) and the *FBXO30* assays (1/13; 7.6%) and in 4 patients with the *HAPLN3* assay (4/102; 3.4%) (Figure 3A–C). Notably, although methylated DNA was detected in 98%–100% of RP tissue specimens (41/41 for *HAPLN3* and *FBXO30,* 40/41 *DOCK2*), methylated ctDNA was not detected in matched plasma samples by *HAPLN3 (0/*41) and *FBXO30 (*0/2) and only in one plasma sample using the *DOCK2* assay (1/41; 2.4%, methylated ctDNA/cfDNA ratio at 0.001) (Figure 3D–F). *FBXO30* was only tested on matching plasma from two patients due to limited cfDNA amounts available. Based on ROC curve analysis, the specificity/sensitivity for PCa in tissue samples was 90%/90% for *DOCK2*, 84%/100% for *HAPLN3*, and 100%/95% for *FBXO30*, with corresponding AUCs of 0.970, 0.992, and 0.972, respectively (Figure 3G–I). This indicates that the tumor DNA in patients with localized PCa is indeed hypermethylated for these candidate markers, but this DNA is in most cases not released/leaking into the circulation in sufficient amounts to allow detection by MS-ddPCR in plasma. Thus, using our three PCa DNA methylation specific markers we were not able to detect methylated ctDNA in plasma from majority of patients with BPH or localized PCa.

### 3.4. Detection of Methylated ctDNA in Plasma Samples from Patients with De Novo mPCa

**Figure 4:**
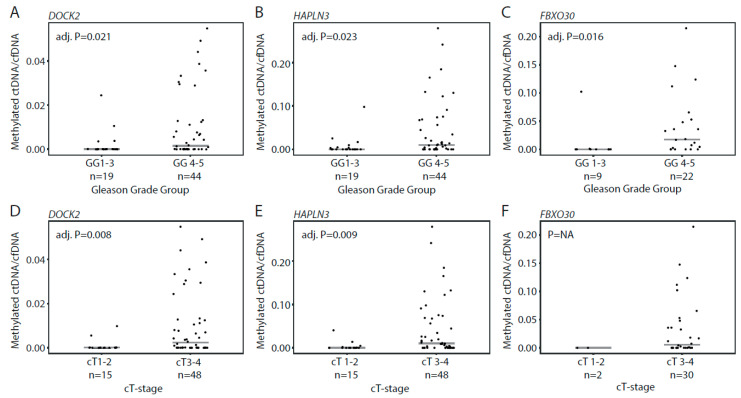
Association between methylated ctDNA fractions in plasma samples from patients with de novo mPCa and clinical parameters. Ratio between methylated ctDNA and cfDNA for *DOCK2* and *HAPLN3* in patients assigned to Gleason Grade group 1–3 vs. 4–5 (**A**–**C**) and clinical T-stage 1–2 vs. 3–4 (**D**–**F**). Bars indicate median values in each group. Each dot represents one analyzed sample. *p*-values were calculated with Mann–Whitney test. Adj. P, Benjamini–Hochberg adjusted *p*-value. GG, Gleason Grade Group. NA, not applicable.

In patients with de novo mPCa, we detected methylated ctDNA in 61.5% using our three assays, with 29/65 (44.6%) patients positive for *DOCK2*, 39/65 (60.0%) positive for *HAPLN3*, and 18/32 (56.3%) positive for *FBXO30* (Figure 3A–C). For all assays, the ctDNA fraction (methylated ctDNA/cfDNA) was significantly higher in Gleason Grade Group 4–5 vs. 1–3 (Mann–Whitney test, adj. *p* = 0.021, 0.023, and 0.016, respectively, Figure 4A–C). Similarly, the ctDNA fraction was significantly higher in cT3-cT4 vs. cT1-cT2 for *DOCK2* and *HAPLN3* (Mann–Whitney test, adj. *p* = 0.008, 0.009, Figure 4D,E), while this could not be assessed for *FBXO30* (only two samples available for analysis in the cT1–2-group; Figure 4F). Thus, in the de novo mPCa patients, high Gleason Grade Group and/or high clinical T-stage seem to be associated with significantly higher ctDNA fractions.

### 3.5. Methylated ctDNA in De Novo mPCa Patients with Low- and High Tumor-Volume

**Figure 5:**
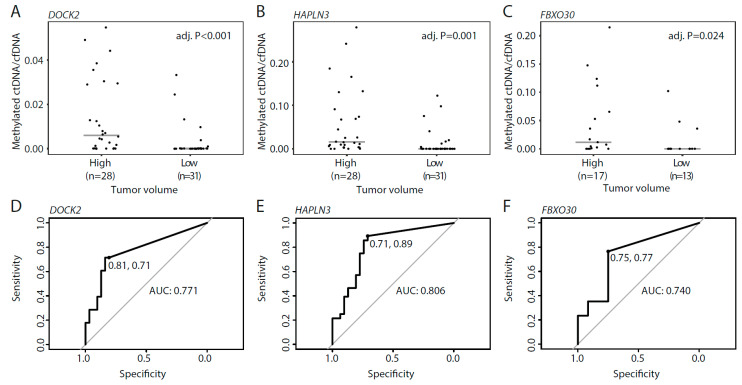
Methylated ctDNA in de novo mPCa patients with high and low tumor volume. (**A**–**C**) Ratio between methylated ctDNA and cfDNA for *DOCK2* (**A**), *HAPLN3* (**B**), and *FBXO30* (**C**) in high and low tumor volume. Each dot represents one analyzed sample. (**D**–**F**) ROC curves comparing high and low tumor volume for *DOCK2* (**D**), *HAPLN3* (**E**), and *FBXO30* (**F**). Horizontal bars indicate median values in each group. *p*-values were calculated using Mann–Whitney tests. Adj. P, Benjamini–Hochberg adjusted *p*-value. ROC, receiver operating curve. AUC, area under the curve.

For patients with de novo mPCa, disease volume determined by imaging is used for treatment selection. Therefore, we wanted to examine whether ctDNA levels in mPCa patients were associated with disease burden (defined by CHAARTED criteria [3]). In mPCa patients with low tumor volume, methylated ctDNA was detected in a total of 32.3% (10/31) of the patients, with 6/31 (19.4%) patients positive for *DOCK2*, 9/31 (29.0%) positive for *HAPLN3*, and 3/12 (25.0%) positive for *FBXO30* (Figure 5A–C). In contrast, methylated ctDNA was detected in 89.2% (25/28) of mPCa patients with high tumor volume; 20/28 (71.4%) patients positive for *DOCK2*, 25/28 (89.2%) positive for *HAPLN3*, and 13/17 (76.5%) positive for *FBXO30* (Figure 5A–C). For all assays, the methylated ctDNA level was significantly increased in high vs. low tumor volume mPCa, indicating that there is a positive association between tumor burden and methylated ctDNA levels in plasma (*p* < 0.024, Mann–Whitney test, Figure 5A–C). Specificity/sensitivity for high tumor volume was 81%/71% for *DOCK2*, 71%/89% for *HAPLN3*, and 75%/77% for *FBXO30* (Figure 5D–F). These results indicate a biomarker potential for methylated ctDNA for the identification of mPCa patients with high tumor volume, which may be utilized in the future for treatment selection at this disease stage.

### 3.6. Methylated ctDNA as a Predictor of Time to mCRPC Progression, PCa-Specific Survival, and Overall Survival

**Figure 6:**
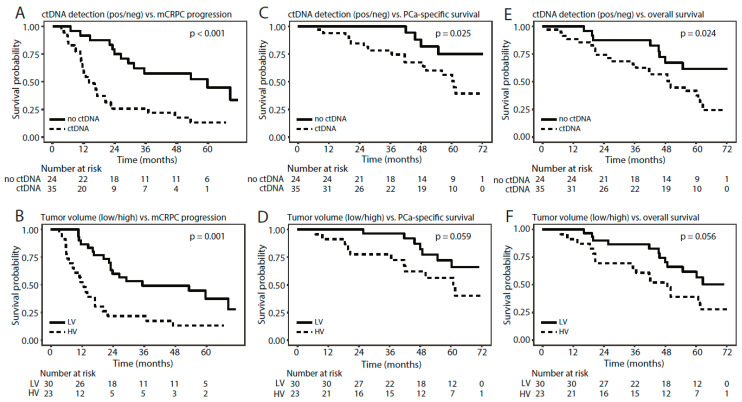
Kaplan–Meier survival analyses for de novo mPCa patients, stratified by (**A**,**C**,**E**) detection of methylated ctDNA (any assay) or by (**B**,**D**,**F**) tumor volume using (**A**,**B**) mCRPC progression, (**C**,**D**) PCa-specific survival, and (**E**,**F**) overall survival as endpoints. *p*-values were calculated using log-rank tests.

In patients with de novo mPCa, we further investigated whether detection of methylated ctDNA could predict time to mCRPC progression. Detection of methylated ctDNA (any of the three assays: *DOCK2/HAPLN3/FBXO30*) was associated with significantly shorter time to mCRPC progression in univariate Cox regression (*p* = 0.001, HR = 3.1 (1.7–6.6), Table 2) and Kaplan–Meier analyses (*p* < 0.001, log-rank test, Figure 6A). High tumor volume was also associated with significantly shorter time to mCRPC progression in both univariate Cox regression (*p* = 0.001, HR = 2.9 (1.5–5.6), Table 2) and Kaplan–Meier analyses (*p* = 0.001, log rank test, Figure 6B). In multivariate Cox regression analyses, detection of methylated ctDNA remained a significant predictor of time to mCRPC progression independent of tumor volume (*p* = 0.012, HR = 3.0 (1.3–6.9), Table 2). Furthermore, detection of methylated ctDNA was associated with significantly shorter PCa-specific and overall survival in both univariate Cox regression (*p* = 0.034/0.029, HR = 3.3 (1.1–9.8)/2.5 (1.1–5.5), Table 2) and Kaplan–Meier analyses (*p* = 0.025 and 0.024, respectively, log-rank test. Figure 6C,E), whereas tumor volume (high/low) was not significantly associated with these endpoints (Table S7 and Figure 6). Due to the limited number of events, multivariate Cox regression analyses were not performed. Similar results were obtained for the assays individually for all three endpoints (Figure S4, Table S7), however, overall the sensitivity for predicting mCRPC progression, PCa-specific survival, and overall survival increased when combining the assays (Table S8).

Thus, de novo mPCa patients with detectable methylated ctDNA had significantly shorter time to mCRPC progression as well as significantly shorter PCa-specific and overall survivals than patients with no detectable methylated ctDNA.

## 4. Discussion

In the present study, we identified three highly PCa specific DNA methylation biomarkers, *DOCK2*, *HAPLN3*, and *FBXO30*, with no false positive signal from PBCs or plasma cfDNA from healthy donors. Using LNA-enhanced methylation specific ddPCR assays for these candidates, we evaluated their biomarker potential in a clinical cohort comprising >250 plasma samples from healthy controls as well as BPH, PCa, and de novo mPCa patients.

CfDNA in the bloodstream mainly originates from the hematopoietic system, but previous studies have observed correlations between higher cfDNA concentrations in plasma and higher disease stage in advanced cancers [37,38,39]. Here, we did not find significant differences in total plasma cfDNA levels between healthy controls (*n* = 12), BPH (*n* = 61), localized PCa (*n* = 102), or mPCa (*n* = 65) patients. Previous reports are conflicting, as some studies have reported significantly higher [40,41,42] and other significantly lower [43,44] plasma cfDNA levels in BPH compared to localized PCa patients. Moreover, in contrast to the results from our patient sample set, two previous studies found higher plasma cfDNA levels in PCa patients with lymph node or distant metastases compared to patients with localized PCa [43,44,45]. Thus, additional studies on larger cohorts are needed to firmly establish whether plasma cfDNA levels have a potential role in PCa diagnosis and risk stratification. Of note, earlier studies have also found significantly higher cfDNA levels in patients with advanced T-stages (T3–4), whereas age, Gleason score, and serum PSA did not correlate with cfDNA levels [41,45]. This is in agreement with our results in which only higher cT-stage was significantly associated with higher cfDNA levels in de novo mPCa patients.

To investigate the abundance of methylated ctDNA in our clinical cohort, we examined DNA methylation of *DOCK2*, *HAPLN3*, and *FBXO30*. We did not detect methylated ctDNA in plasma samples from healthy controls (*n* = 12). However, both the *HAPLN3* and the *FBXO30* assay detected methylated ctDNA in 1 BPH patient (1/61), and this patient died (unknown reason) within one year after plasma sampling. Methylated ctDNA was undetectable in plasma samples from the majority of patients with localized (defined by imaging) PCa (*n* = 102), but five patients had few detectable copies of methylated ctDNA (methylated ctDNA/cfDNA ratio below 0.002). It can be questioned whether three of these patients had localized PCa at inclusion as one patient had PSA above 600 ng/mL at inclusion, another had increasing PSA values after RP, and a third patient had localized advanced disease (cT3b) with Gleason Score 9. Notably, in patients where the DNA methylation assays were positive in RP tissue specimens, no signal was detected in matching plasma samples with *HAPLN3* and FBXO30 assay and only in plasma from one patient with the *DOCK2* assay. The general lack of signal in matched plasma samples is unlikely to be explained by too small plasma sample volumes, as we on average extracted cfDNA from 8.9 mL plasma (range: 2.6–20 mL) and, furthermore, used an average input of 6538 bisulfite converted copies per duplex MS-ddPCR reaction (range: 832–29,524). Rather it indicates that the plasma ctDNA amount is below our detection limit (16, 16, and 32 genome equivalents in a background of ≤20,000 genome equivalents for *DOCK2*, *HAPLN3*, and *FBXO30*, respectively).

The general lack of methylated ctDNA signal in patients with localized PCa in our clinical cohort is in accordance with a recent study investigating somatic copy number alterations and mutations in pre-RP plasma cfDNA from 112 PCa patients using ultra-low-pass whole-genome sequencing and targeted resequencing [46]. In this study, none of the sequencing strategies detected ctDNA in plasma samples from patients with localized PCa, nor in high-risk patients who subsequently experienced biochemical recurrence. However, ctDNA was detectable by both sequencing approaches in plasma samples from 4 out of 7 (57%) mPCa patients, which is similar to our results. Together, this indicates that the amount of DNA released from localized PCa tumors into plasma is very low. Although our three methylation-specific assays are very sensitive (detection of minimum 32 genome equivalents in a background of ≤20,000 genome equivalents), combining several markers as e.g., in cfMeDIP-seq [47], has been suggested to increase the sensitivity for methylation-based ctDNA detection considerably. In line with this, combining our markers into one variable (detectable methylated ctDNA by any assay vs. no detectable methylated ctDNA), increased the sensitivity for *DOCK2* and *HAPLN3* for both mCRPC progression and PCa-specific survival in de novo mPCa patients. For all three endpoints the sensitivity for the *FBXO30* assay alone was higher than the combined variable, but the sensitivity of *FBXO30* was only based on analysis of 26 de novo mPCa patients compared to 59 de novo mPCa patients for *DOCK2*, *HAPLN3,* and the combined variable. These results are in line with previous studies, however limited by the few number of patients. Further studies on larger cohorts are needed to validate our preliminary findings. Future studies are also needed to investigate if it is possible to detect methylated ctDNA in localized PCa using >3 DNA methylation markers.

In patients with de novo mPCa, we detected methylated ctDNA in 61.5% of the plasma samples and observed a positive correlation between ctDNA abundance (i.e., ratio between methylated *DOCK2/HAPLN3* ctDNA and total cfDNA, respectively), Gleason Grade Group and clinical T-stage at diagnosis indicating that the level of ctDNA in de novo mPCa patients increase with increasing tumor burden. However, it should be noted that our results are limited by the small number of mPCa patients in our cohort and warrants further validation in larger cohorts.

In de novo mPCa patients, we detected methylated ctDNA in 32.3% of the patients with low tumor volume and in 89.3% of patients with high tumor volume using the *DOCK2*, *HAPLN3*, and *FBXO30* assays (positive for at least one assay). These findings correspond to a previous study on patients with de novo mPCa, where 74.3% (26/35) of the examined patients had measureable ctDNA prior to ADT, as detected by somatic point mutation and copy number alterations (targeted sequencing of 73 PCa driver genes) [19]. Moreover, we also found that detection of methylated ctDNA was associated with significantly shorter time to mCRPC progression, independent of tumor volume. These results indicate that detection of methylated ctDNA in mPCa patients could potentially be utilized in the future for treatment selection as a predictor of risk, and thus identify patients who would benefit from intensified treatment, e.g., combination therapies with ADT and docetaxel, abiraterone, or apalutamide. Currently, treatment decisions for adjuvant treatment at mPCa diagnosis are solely based on traditional image-based disease burden stratification (high- and low tumor volume) and does not consider the molecular landscape incl. DNA methylation. Inclusion of DNA methylation in patient stratification could provide an opportunity for a more personalized treatment selection which may improve time to mCRPC progression. Furthermore, the results presented here also indicate that detection of methylated ctDNA is associated with significantly shorter PCa-specific—and overall survival. Patient stratification based on tumor volume (high vs. low) did not result in significant differences in PCa-specific and overall survival in the present patient cohort. These results further indicate a potential role for methylated ctDNA in patient stratification. Additional studies on large independent cohorts with long follow-up are needed to evaluate the possible clinical utility of methylated ctDNA as a predictive biomarker in mPCa patients. Future studies should also examine whether other clinical variables besides tumor volume are associated with detection of methylated ctDNA in mPCa, as the results in this study are limited by few events.

In conclusion, we identified three biomarkers (*DOCK2*, *HAPLN3*, and *FBXO30)* specifically hypermethylated in PCa, but not in PBCs or plasma cfDNA from healthy controls. Although methylated ctDNA was generally not detected in patients with localized PCa for the three markers, we detected methylated ctDNA in a total of 61.5% of patients with de novo mPCa for at least one of the three markers. Interestingly, methylated ctDNA was more frequently detected in de novo mPCa patients with high tumor volume compared to low tumor volume suggesting that methylated ctDNA may also be used to identify mPCa patients who will benefit from intensified treatment. Another important finding in this study is that methylated ctDNA seems to be an independent predictor of time to mCRPC progression further indicating that methylated ctDNA may be used to guide treatment selection in mPCa patients.

## Figures and Tables

**Table 1:** Patient characteristics of clinical cohort.

	Metastatic PCa (*n* = 65)	Localized PCa (*n* = 102)	Benign Prostatic Hyperplasia (*n* = 61)	Healthy Controls (*n* = 36)
Age (years): median (min-max)	72 (47–86)	66 (43–81)	71 (49–88)	> 45
PSA (ng/mL): median	42.0	9.4	4.3	NA
(min-max)	(1.9–1171)	(1.2–812)	(0.5–42.8)	
cT-stage: *n* (%)				
cT1–2	15 (23)	82 (80)	NA	NA
cT3–4	48 (74)	17 (17)	NA	NA
Unknown	2 (3)	3 (3)	NA	NA
Gleason Grade Group: *n* (%)				
I	3 (5)	2 (2)	NA	NA
II	6 (9)	69 (67)	NA	NA
III	10 (15)	20 (20)	NA	NA
IV	13 (20)	2 (2)	NA	NA
V	31 (48)	6 (6)	NA	NA
Unknown	2 (3)	3 (3)	NA	NA
Regional lymph node metastasis status: *n* (%)				
N1	15 (23)	4 (4)	NA	NA
N0	6 (9)	27 (26)	NA	NA
NX	1 (2)	53 (52)	NA	NA
Unknown	43 (66)	18 (18)	NA	NA
Metastasis status: *n* (%)				
M0	0 (0)	102 (100)	NA	NA
M1	65 (100)	0 (0)	NA	NA
Metastasis location/volume: *n* (%)				
M1–lymph node only (non regional)	4 (6)	NA	NA	NA
M1–Low tumor volume	31 (48)	NA	NA	NA
M1–High tumor volume	28 (43)	NA	NA	NA
Unknown	2 (3)	NA	NA	NA
Treatment after diagnosis: *n* (%)				
RP	0 (0)	90 (88)	NA	NA
RT	0 (0)	3 (3)	NA	NA
Active surveillance	0 (0)	1 (1)	NA	NA
Antiandrogen blockage (Bicalutamide)	0 (0)	2 (2)	NA	NA
Androgen deprivation therapy	65 (100)	6 (6)	NA	NA
Biochemical recurrence (BCR) after RP: *n* (%)				
BCR	NA	19 (21)	NA	NA
No BCR	NA	67 (75)	NA	NA
Unknown	NA	4 (4)		
Plasma volume (mL): Mean (min-max)	6.8 (1.4–19.9)	8.9 (2.6–20.0)	10.2 (6.5–19.7)	6.7 (4.7–7.7)
cfDNA concentration (ng/mL plasma): Median (min-max)	6.5	5.6	6.0	6.0
	(2.0–196.4)	(2.0–47.3)	(2.7–35.7)	(2.1–10.3)

**Table 2:** Univariate and multivariate Cox regression using time to metastatic castration-resistant PCa (mCRPC) progression, PCa-specific, and overall survival as endpoints. Detection of methylated ctDNA was defined as detection by at least one assay.

Variable	Characteristics	Univariate			Multivariate		
		HR (95% CI)	*p*-val	C-index	HR (95% CI)	*p*-val	C-index
**Endpoint: mCRPC**							
Methylated ctDNA	No ctDNA vs. ctDNA	3.1 (1.7–6.6)	0.001	0.654	3.0 (1.3–6.9)	0.012	0.715
Tumor volume	Low vs. high	2.9 (1.5–5.6)	0.001	0.657	1.8 (0.8–3.8)	0.202	
**Endpoint: PCa-Specific Survival**							
Methylated ctDNA	No ctDNA vs. ctDNA	3.3 (1.1–9.8)	0.034	0.639	-	-	-
Tumor volume	Low vs. high	2.4 (0.94–6.2)	0.068	0.636	-	-	-
**Endpoint: Overall Survival**							
Methylated ctDNA	No ctDNA vs. ctDNA	2.5 (1.1–5.5)	0.029	0.597	-	-	-
Tumor volume	Low vs. high	2.1 (0.97–4.3)	0.060	0.605	-	-	-

## Supplementary Materials

The following are available online at https://www.mdpi.com/2073-4409/9/6/1362/s1; Figure S1-part1: In silico analyses of 27 biomarker candidates using the Marmal-aid database; Figure S1-part2: In silico analyses of 27 biomarker candidates using the Marmal-aid database; Figure S2-part1: Small-scale experimental validation using quantitative methylation-specific PCR (qMSP); Figure S2-part2: Small-scale experimental validation using quantitative methylation-specific PCR (qMSP); Figure S3: Association between cfDNA abundance and clinicopathological parameters in the clinical cohort; Figure S4: Kaplan–Meier survival analyses in de novo mPCa patients for *DOCK2*, *HAPLN3*, and *FBXO30* assays individually; Table S1: Overview of samples used for small-scale experimental evaluation (qMSP), technical evaluation (MS-ddPCR), and the clinical cohort (tissue); Table S2: Overview of plasma extraction and bisulfite conversion efficiency of plasma samples; Table S3: Primers and probes used for qMSP; Table S4: Reference numbers (Qiagen) for LNA primers and probes used for MS-ddPCR; Table S5: Minimum Information for Publication of Quantitative Digital PCR Experiments (dMIQE) guidelines; Table S6: Sensitivity and specificity for the 24 assays subjected to small-scale evaluation using quantitative methylation-specific PCR (qMSP); Table S7: Cox regression analyses of *DOCK2*, *HAPLN*3, and *FBXO30* using time to mCRPC, PCa-specific survival, and overall survival as endpoints; Table S8: Sensitivity for prediction of mCRPC progression, PCa-specific survival, and overall survival for *DOCK2*, *HAPLN3*, *FBXO30*, and ctDNA positive (by any of the three assays) vs. negative in de novo mPC patients.

## Abbreviations

450K	Infinium Human Methylation array 450
AN	Adjacent normal
AUC	Area under the curve
BCR	Biochemical recurrence
BPH	Benign prostatic hyperplasia
cfDNA	Circulating cell-free DNA
ddPCR	Droplet digital polymerase chain reaction
FFPE	Formalin-fixed paraffin-embedded
LNA	Locked nucleic acids
mCRPC	Metastatic castration-resistant prostate cancer
mPCa	Metastatic prostate cancer
MS-ddPCR	Methylation-specific droplet digital polymerase chain reaction
NA	Not available
NS	Not significant
OS	Overall survival
PBC	Peripheral blood cells
PBMC	Peripheral blood mononuclear cells
PCa	Prostate cancer
qMSP	Quantitative methylation-specific polymerase chain reaction
ROC	Receiver operating characteristic
RP	Radical prostatectomy
TUR-P	Transurethral resection of the prostate
