# Supplementary material for: Epigenetic Analysis of Circulating Tumor DNA in Localized and Metastatic Prostate Cancer: Evaluation of Clinical Biomarker Potential
Source: Cells. 2020 May 31;9(6):1362. doi: 10.3390/cells9061362 (PMC7349912; doi:10.3390/cells9061362)
Supplement: Supplementary file 1 [file cells-09-01362-s001.pdf]

Supplementary figures

Figure S1-part1: In silico analyses of 27 biomarker candidates using the Marmal-aid database. The candidates were identified in previous studies: Bjerre *et al.* [14] (*C2orf88, C2orf43, cg12799885, DOCK2, FBXO30-cg09094393, FBXO30-cg23095612, GRASP, HIF3A, MOB3B, PFKP, TPM4)*, Strand *et al. [12]* (*COL4A6, CYBA, HLF, LOC149113, LRRC4, PROM1, RHCG, TCAF1)*; Haldrup *et al.* [10] (*AOX1, C1orf114, HAPLN3, KLF, ST6GALNAC3, ZNF660*); Kristensen *et al.* [7] (*GABRE*); and Goering *et al.* [26] (*GSTP1*).

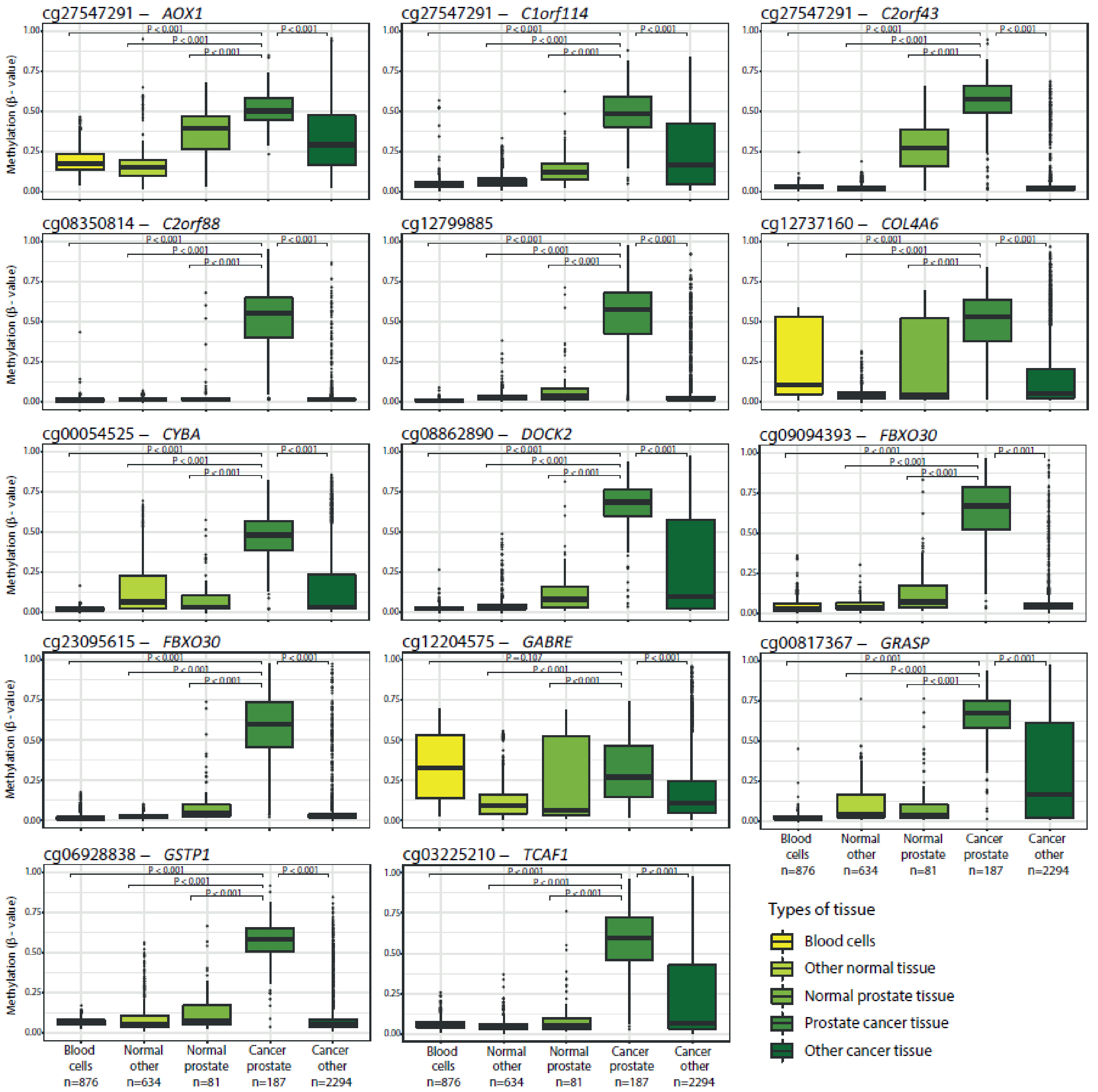

Figure S1-part2: In silico analyses of 27 biomarker candidates using the Marmal-aid database. The candidates were identified in previous studies: Bjerre *et al.* [14] (*C2orf88, C2orf43, cg12799885, DOCK2, FBXO30-cg09094393, FBXO30-cg23095612, GRASP, HIF3A, MOB3B, PFKP, TPM4), Strand et al. [12]* (*COL4A6, CYBA, HLF, LOC149113, LRRC4, PROM1, RHCG, TCAF1)*; Haldrup *et al.* [10] (*AOX1, C1orf114, HAPLN3, KLF, ST6GALNAC3, ZNF660*); Kristensen *et al.* [7] (*GABRE*); and Goering *et al.* [26] (*GSTP1*).

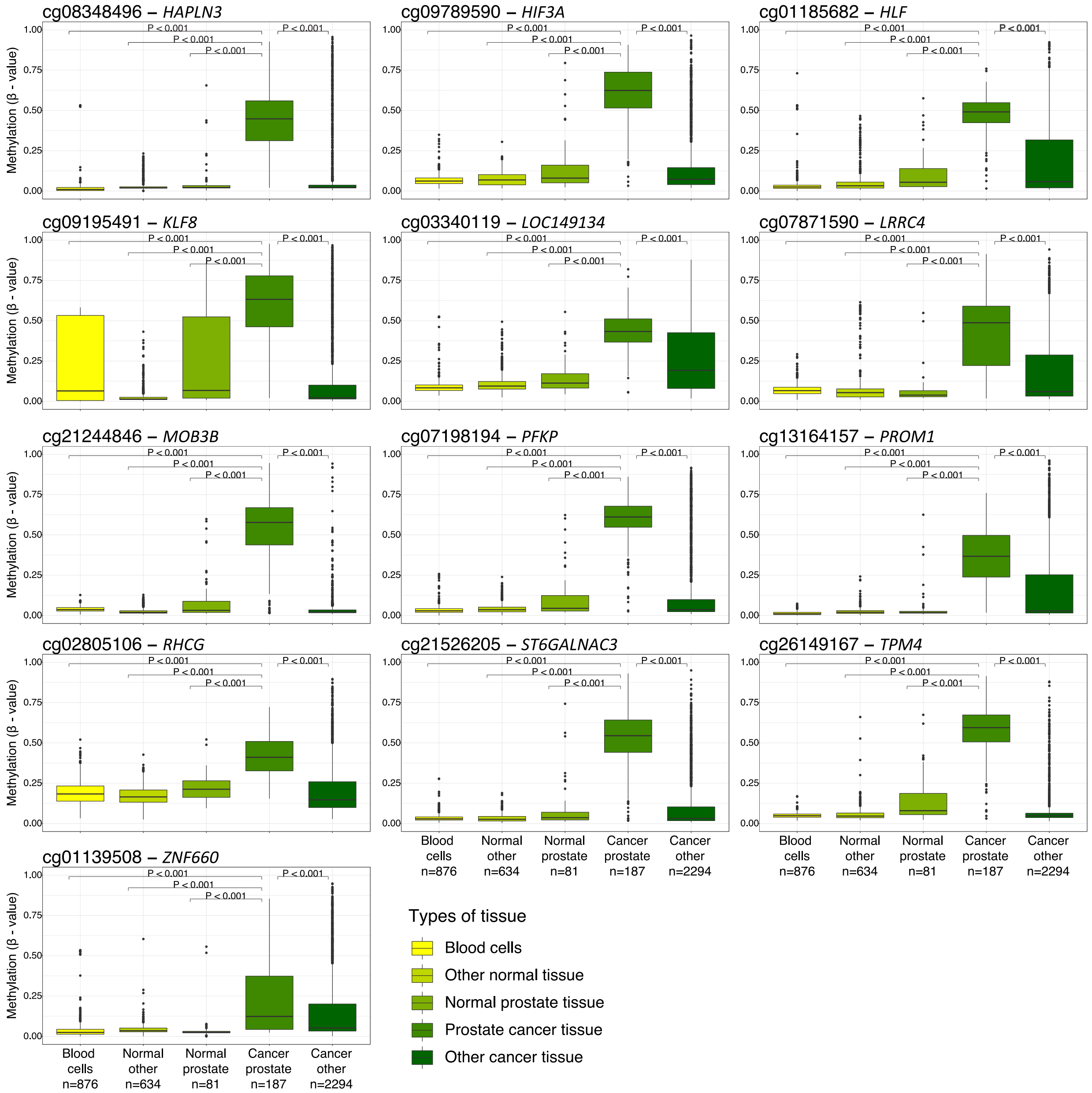

Figure S2-part1: Small-scale experimental validation using quantitative methylation-specific PCR (qMSP)

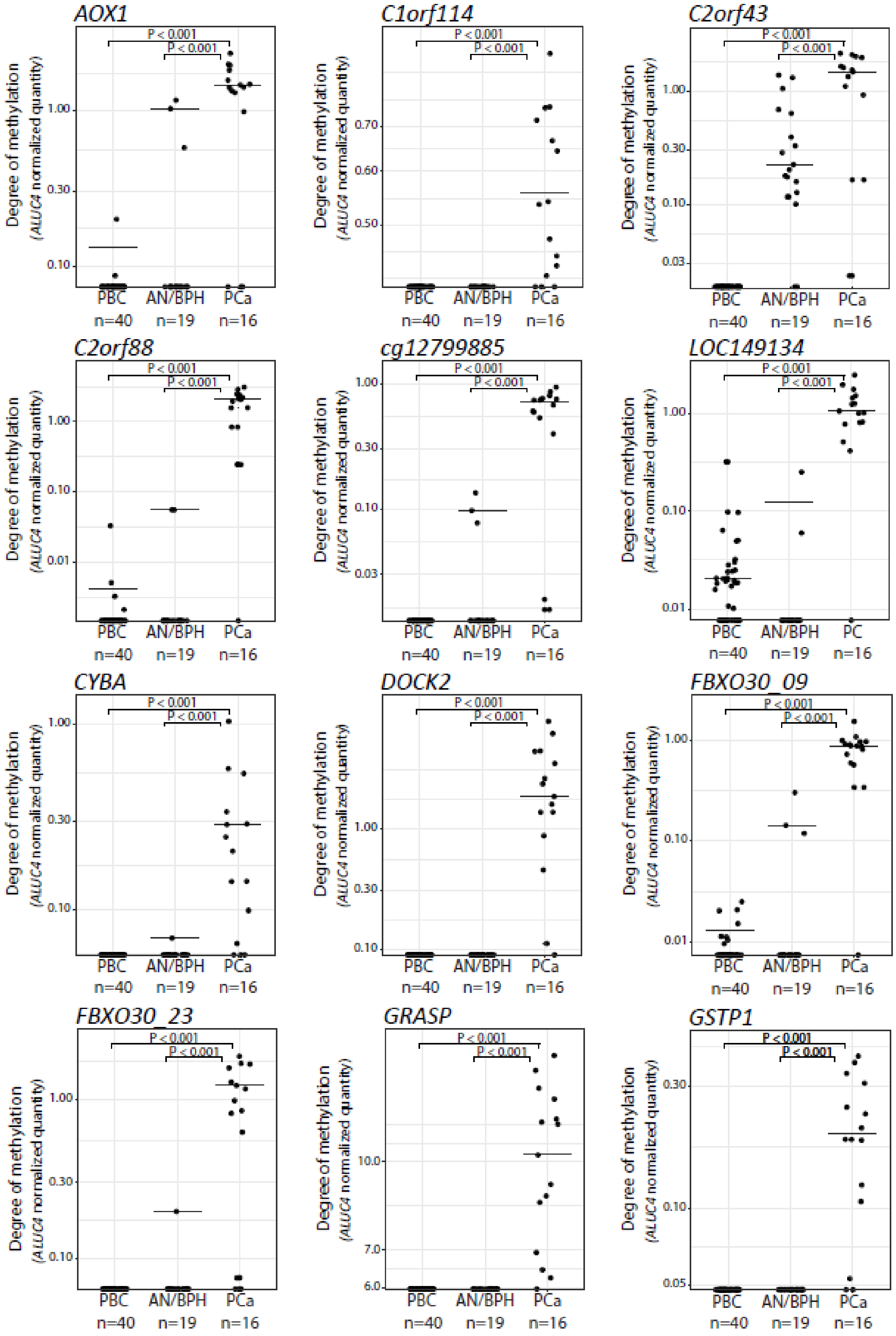

Figure S2-part2: Small-scale experimental validation using quantitative methylation-specific PCR (qMSP)

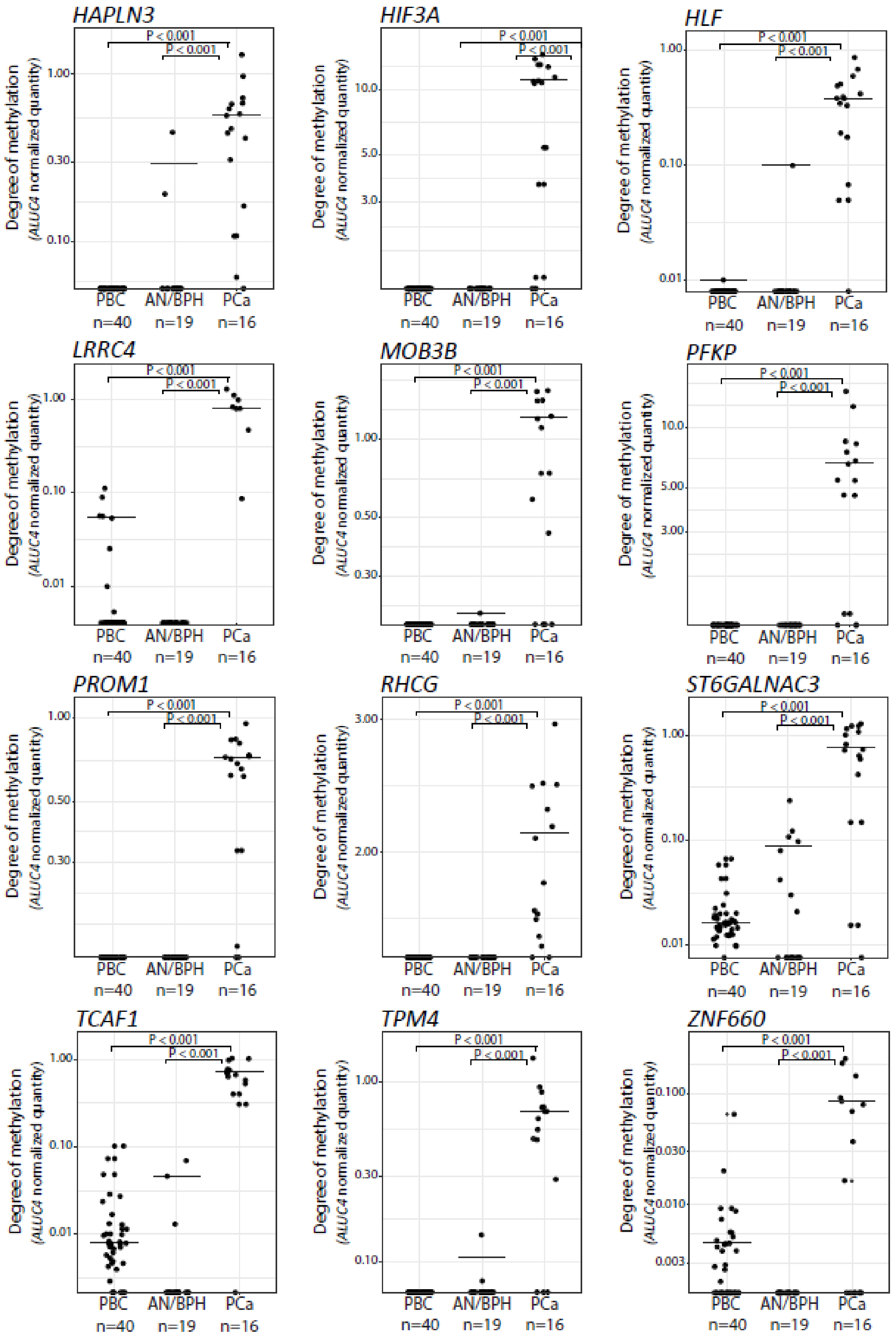

Figure S3: Association between cfDNA concentration and clinicopathological parameters in the clinical cohort (see also Table 1). P values from Mann-Whitney test (A, C-E) or spearman's rho test (B). NS, not significant.

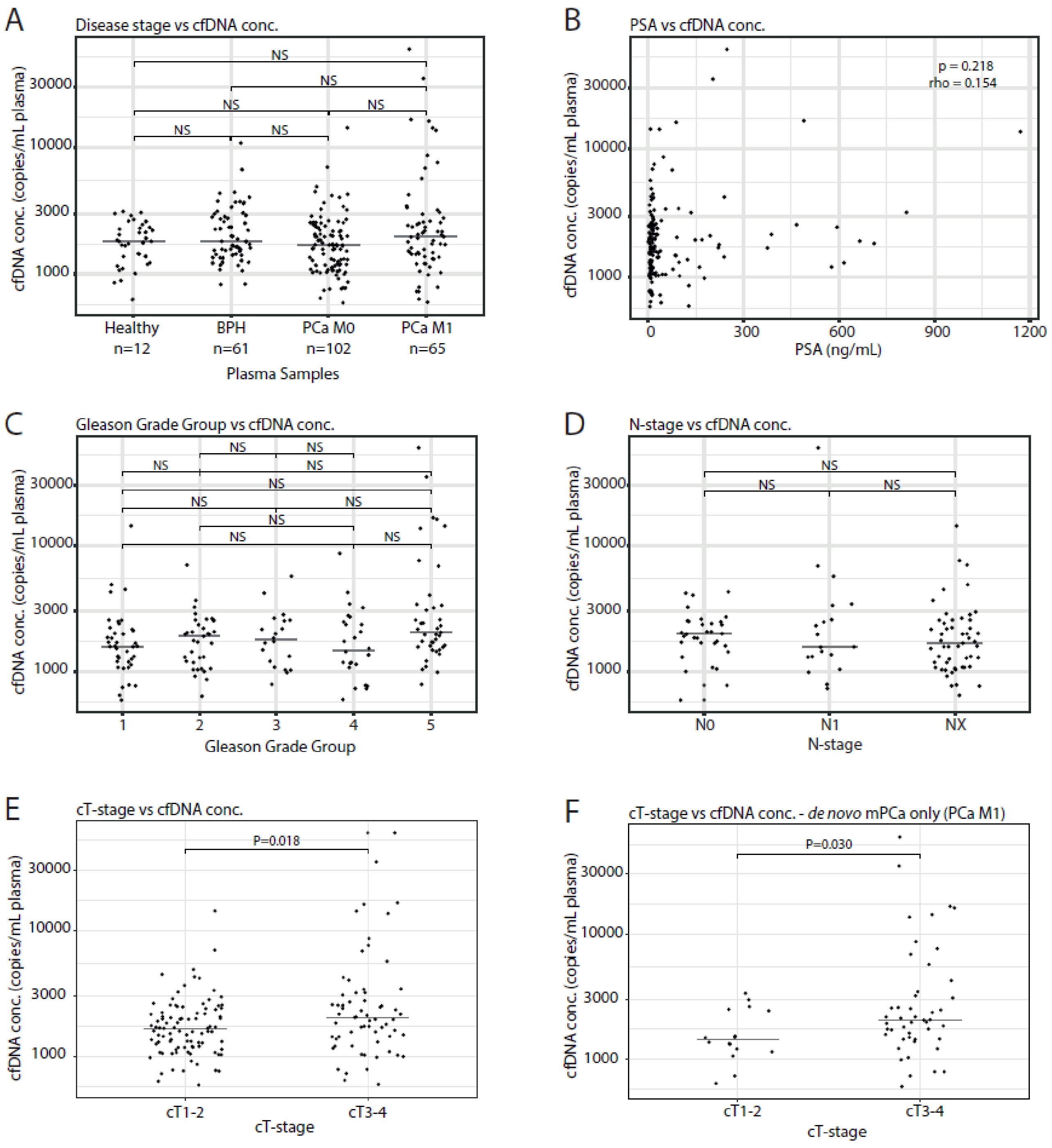

Figure S4: Kaplan-Meier survival analyses in *de novo* mPCa patients for *DOCK2*, *HAPLN3*, and *FBXO30* assays individually. Patients were stratified by detection of methylated ctDNA based on our assays *DOCK2 (**A, E, G**)*, *HAPLN3 (**B, F, H**)*, and *FBXO30 (**C, G, I**)* using time to mCRPC (**A-C**), PCa-specific survival (**D-F**), or overall survival (**G-I**) as end-points. P-values were calculated using log-rank tests.

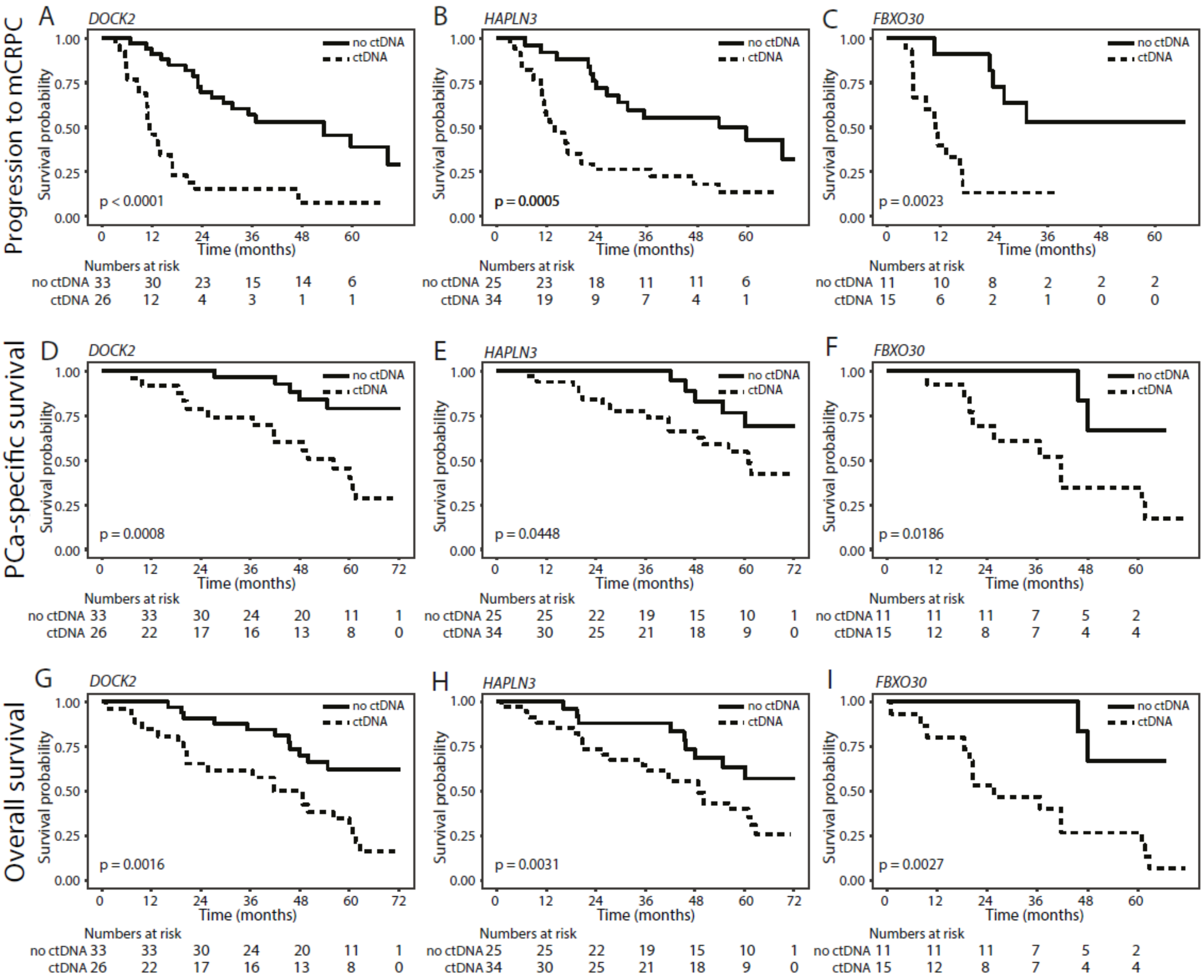

Table S1: Overview of samples used for small-scale experimental evaluation (qMSP), technical evaluation (MS-ddPCR), and the clinical cohort (matched tissue and plasma (MS-ddPCR)).

	Small-scale experimental evaluation (qMSP)					Technical evaluation (MS-ddPCR)		Clinical cohort - Matched tissue and plasma (MS-ddPCR)	
Samples	PCa (n=20)	AN (n=13)	BPH (n=7)	PBC (n=20)	PBC (n=20)	PBC (n=52)	Healthy blood donors (n=70)	PCa (n=55)	AN (n=51)
Material	FFPE tissue, RP punch biopsy	FFPE tissue, RP punch biopsy	FFPE tissue, TUR-P punch biopsy	Whole blood	Buffy coat	Buffy coat	Plasma	FFPE tissue, RP punch biopsy	FFPE tissue, RP punch biopsy
Volume (mL), median (min-max)	-	-	-	-	-	-	7.7 (5.9-7.7)	-	-
Age (years), median (min-max)	66 (56-71)	61 (57-79)	70 (57-81)	67 (56-71)	57 (51-63)	57 (51-67)	< 45	64 (48-75)	64 (48-75)
Samples BS converted, n (%)	20 (100)	13 (100)	7 (100)	20 (100)	20 (100)	46 (88)	67 (95)	48 (87)	36 (71)
Samples analysed, n (%)	16 (80)	13 (100)	6 (86)	20 (100)	20 (100)	44 (85)	64 (91)	41 (75)	11 (22)

qMSP, quantitative methylation-specific PCR. MS-ddPCR, methylation specific droplet digital PCR. PCa, prostate cancer. AN, adjacent normal. BPH, benign prostatic hyperplasia. PBC, peripheral blood cells. FFPE, formalin-fixed paraffin-embedded. RP, radical prostatectomy. TUR-P, transurethral resection of the prostate. BS, bisulfite.

Table S2: Overview of plasma extraction and bisulfite conversion efficiency of plasma samples

	Technical evaluation (MS-ddPCR)	Clinical cohort (MS-ddPCR)			
**Samples**	**Healthy blood donors (n=70)**	**Healthy male blood donors (n=36)**	**BPH (n=61)**	**Localized PCa (n=102)**	**mPCa (n=68)**
**Extraction efficiency, mean (1st-3th quartile)**	88 (75-100)	83 (73-93)	89 (78-99)	73 (64-83)	71 (59-82)
**cfDNA concentration (copies/mL plasma), median (min-max)**	2149 (606-5086)	1814 (629-3116)	1811 (827-10823)	1693 (593-14321)	1966 (599-59550)
**Samples BS converted, n (%)**	67 (95)	36 (100)	61 (100)	102 (100)	67 (100)
- median cfDNA copies before BS (min-max)	16335 (4099-41402)	12266 (4871-20731)	14600 (6468-207570)	11458 (2649-78355)	8131 (1910-708730)
- Recovery after BS conversion (%), median (min-max)	46(28-66)	56 (39-86)	48 (30-77)	57 (8-99)	58 (33-96)
- Median bisulfite converted cfDNA copies per duplex ddPCR reaction (min-max)	4936 (1445-9900)	3762 (1495-6237)	4734 (1548-88814)	4290 (832-29524)	2710 (752-394460)
**Samples included in final analyses, n (%)**	64 (91)	36 (100)	61 (100)	102 (100)	65 (97)

MS-ddPCR, methylation specific digital droplet PCR. BPH, benign prostatic hyperplasia. PCa, prostate cancer. mPCa, metastatic prostate cancer. BS, bisulfite.

Table S3: Primers and probes used for qMSP.

Submitted as an excel-file

Table S4: Reference numbers (Qiagen) for LNA primers and probes used for MS-ddPCR.

Submitted as an excel-file

Table S5: Minimum Information for Publication of Quantitiative Digital PCR Experiments (dMIQE) guidelines.

Submitted as an excel-file

Table S6: Sensitivity and specificity for the 24 candidate markers subjected to small-scale evaluation using quantitative methylation-specific PCR (qMSP). Sensitivity, specificity, negative and positive predictive values were calculated based on analyses of 16 PCa tissue versus 19 AN/BPH tissue samples. False detection rates are reported for 40 PBC samples analysed. Black highlight: assays that were selected for further validation. Red highlight: parameter by which the assays were excluded from further analyses. Blue highlights: the three top candidate markers selected for the final analyses.

Small-scale evaluation (qMSP)					
Assay	Sensitivity	Specificity	Negative predictive value	Positive predictive value	False detection rate for PBC
AOX1	0.81	0.84	0.84	0.81	0.05
C1orf114	**0.75**	**1.00**	**0.83**	**1.00**	**0.00**
C2orf43	0.94	0.11	0.67	0.47	0.00
C2orf88	0.94	0.89	0.94	0.88	0.10
**cg12799885**	**0.94**	**0.84**	**0.94**	**0.83**	**0.00**
CYBA	**0.81**	**0.95**	**0.86**	**0.93**	**0.00**
DOCK2	0.94	1.00	0.95	1.00	0.00
FBXO30-cg09094393	0.94	0.84	0.94	0.83	0.20
FBXO30-cg23095612	0.75	0.95	0.82	0.92	0.00
GRASP	**0.94**	**1.00**	**0.95**	**1.00**	**0.00**
GSTP1	**0.88**	**1.00**	**0.90**	**1.00**	**0.00**
HAPLN3	0.94	0.89	0.94	0.88	0.00
HIF3A	**0.88**	**1.00**	**0.90**	**1.00**	**0.00**
HLF	0.94	0.95	0.95	0.93	0.03
LOC149134	0.94	0.89	0.94	0.88	0.60
LRRC4	0.81	1.00	0.86	1.00	0.20
MOB3B	**0.75**	**0.95**	**0.82**	**0.92**	**0.00**
PFKP	**0.83**	**1.00**	**0.86**	**1.00**	**0.00**
PROM1	**0.83**	**1.00**	**0.86**	**1.00**	**0.00**
RHCG	**0.83**	**1.00**	**0.86**	**1.00**	**0.00**
ST6GALNAC3	0.88	0.58	0.85	0.64	0.10
TCAF1	0.88	0.84	0.89	0.82	0.85
TPM4	**0.81**	**0.95**	**0.86**	**0.93**	**0.00**
ZNF660	0.75	1.00	0.83	1.00	0.98

Table S7: Cox regression analyses of *DOCK2*, *HAPLN3*, and *FBXO30* using time to mCRPC, PCa-specific survival, and overall survival as endpoints.

Variable	Characteristics	Univariate		Multivariate		Multivariate		Multivariate	
		HR (95% CI)	p-val	HR (95% CI)	p-val	HR (95% CI)	p-val	HR (95% CI)	p-val
Endpoint: mCRPC									
DOCK2	No ctDNA vs. ctDNA	**4.2 (2.2-8.0)**	**<0.001**	**4.9 (2.2-11.3)**	**<0.001**	-	-	-	-
HAPLN3	No ctDNA vs. ctDNA	**3.1 (1.6-6.1)**	**0.001**	-	-	**2.8 (1.2-6.5)**	**0.024**	-	-
FBXO30	No ctDNA vs. ctDNA	**4.7 (1.6-13.7)**	**0.005**	-	-	-	-	**28.3 (3.2-250.4)**	**0.003**
Tumor volume	Low vs high	**2.9 (1.5-5.6)**	**0.001**	1.3 (0.6-2.9)	0.459	1.8 (0.8-3.8)	0.141	0.9 (0.3-2.7)	0.874
Endpoint: PCa-specific survival									
DOCK2	No ctDNA vs. ctDNA	4.8 (1.7-13)	0.003	-	-	-	-	-	-
HAPLN3	No ctDNA vs. ctDNA	2.7 (1.1-8.3)	0.034	-	-	-	-	-	-
FBXO30	No ctDNA vs. ctDNA	5.2 (1.1-24.1)	0.034	-	-	-	-	-	-
Tumor volume	Low vs high	2.4 (0.93-6.2)	0.068	-	-	-	-	-	-
Endpoint: Overall survival									
DOCK2	No ctDNA vs. ctDNA	3.1 (1.5-6.4)	0.003	-	-	-	-	-	-
HAPLN3	No ctDNA vs. ctDNA	2.3 (1.1-5.0)	0.035	-	-	-	-	-	-
FBXO30	No ctDNA vs. ctDNA	7.1 (1.6-32.0)	0.010	-	-	-	-	-	-
Tumor volume	Low vs high	2.1 (0.97-4.3)	0.060	-	-	-	-	-	-

Table S8: Sensitivity for prediction of mCRPC progression, PCa-specific surivival, and overall survival for *DOCK2, HAPLN3*, *FBXO30*, and ctDNA positive (by any of the three assays) vs. negative in *de novo* mPC patients

End-point	Assay	Sensitivity	Specificity
mCRPC progression	*DOCK2*	54.8%	82.4%
	HAPLN3	66.7%	64.7%
	FBXO30	72.2%	75.0%
	ctDNA pos. (any assay) vs. neg.	69.0%	64.7%
PCa-specific survival	*DOCK2*	65.6%	81.5%
	HAPLN3	75.0%	51.3%
	FBXO30	83.3%	64.3%
	ctDNA pos. (any assay) vs. neg.	80.0%	51.3%
Overall survival	*DOCK2*	75.0%	71.8%
	HAPLN3	71.9%	59.3%
	FBXO30	87.5%	90.0%
	ctDNA pos. (any assay) vs. neg.	75.0%	59.3%
